# Endobronchial metallic foreign body in a Nigerian child: management difficulties and the need for caution: a case report

**DOI:** 10.4076/1757-1626-2-7766

**Published:** 2009-09-15

**Authors:** Adeyi A Adoga, Daniel D Kokong, Nuhu D Ma’an

**Affiliations:** 1 Department of Surgery, Otorhinolaryngology Unit, Jos University Teaching, HospitalPMB 2076, Jos, Plateau StateNigeria; 2 Department of Ear, Nose and Throat Surgery, Federal Medical CenterGombe, Gombe StateNigeria

## Abstract

**Introduction:**

Endobronchial metallic foreign bodies are serious injuries best treated by rigid bronchoscopy as quickly as possible to avoid life threatening respiratory sequelae.

**Case presentation:**

We report the case of a 13-year-old male Nigerian child of the tangale ethnic group who aspirated a metallic foreign body, highlighting the “difficulties” encountered in managing this patient.

**Conclusions:**

There is a need to adequately equip our hospitals for the management of this otherwise straight forward case and alleviate the sufferings of our people. Parents and guardians should exercise caution in the handling of their children/wards.

## Introduction

Foreign body aspirations constitute a reasonable percentage of respiratory emergencies in the pediatric age group [1], causing morbidity and mortality [2].The current mortality rate from foreign body aspiration according to a reported study is between 0 to 1.8% [3].Endobronchial metallic foreign bodies occur less frequently as compared to organic foreign bodies [4]. They constitute serious injuries and their early detection and removal is pertinent to avoid life threatening respiratory sequele such as atelectasis, pneumonia, pulmonary hyperinflation and pneumomediastinum [5] to mention a few.The presenting features are cough, dyspnea, wheezing and fever [4].

The management of aspirated foreign bodies is still by radiological means and the use of bronchoscopes either rigid or flexible fibreoptic. In recent times however, the management of pediatric foreign bodies has become refined both from a diagnostic and therapeutic standpoint [6] with new techniques like helical computerized tomographic (CT) virtual bronchoscopy being used in the evaluation of children with suspected aspiration of foreign bodies [7]. Endobronchial foreign bodies can be very difficult to remove depending on the type and location of the foreign body (they preferentially lodge in the right main bronchus), the experience of the bronchoscopist and the availability of the appropriate instruments for removal [8].

Reduction of these injuries by prevention can be achieved via parental education and avoidance of objects that produce the greatest risk [9].While newer techniques are being developed in the management of tracheobronchial foreign bodies, Nigeria like some other developing nations of the world is still grappling with the non-availability of even the basic diagnostic and therapeutic instruments for the management of these conditions.

In this case report, we present the difficulties encountered in the management of a Nigerian child with an endobronchial radio-opaque foreign body, highlighting the need for properly equipping our health facilities and the exercise of caution by parents and guardians in the handling of their children/wards.

## Case presentation

A 13-year-old male Nigerian child of the tangale ethnic group was referred from the Ear, Nose and Throat Department of the Federal Medical Center, Gombe to the Ear, Nose and Throat Unit of the Jos University Teaching Hospital on account of an aspirated endobronchial radio-opaque foreign body he had placed in his mouth while playing in school. This referral was due to the unavailability of appropriate instruments in the referring hospital for the proper management of this patient.

**Figure 1.**
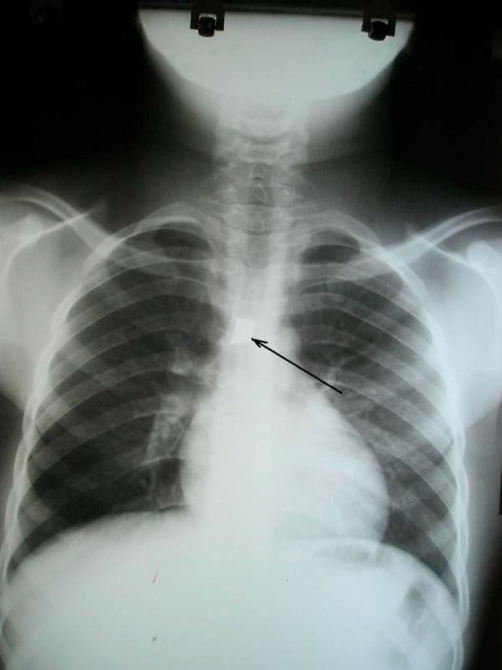
Anterior-posterior chest X-ray showing the radio-opaque foreign body in right main bronchus (Arrow).

**Figure 2.**
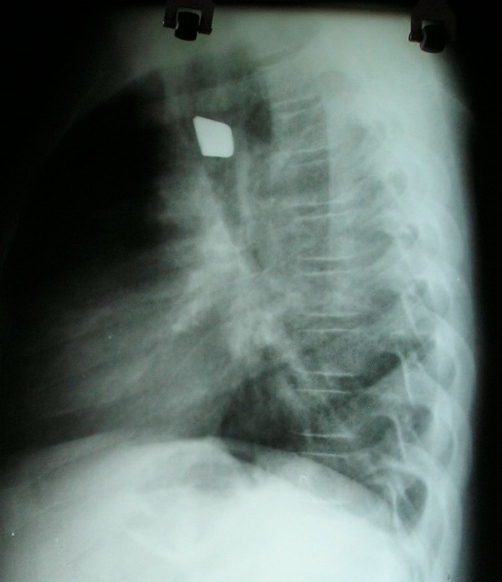
Lateral chest X-ray showing radio-opaque foreign body.

He presented to us 2 days following aspiration, examined and found to be calm and not dyspnoeic. Chest examination revealed decreased air entry in the right hemithorax. Chest X-ray (anterior-posterior and lateral views) taken at the referring hospital confirmed the presence of a rhomboid shaped radio-opaque object in the right main bronchus (Figures 1 and 2).

**Figure 3.**
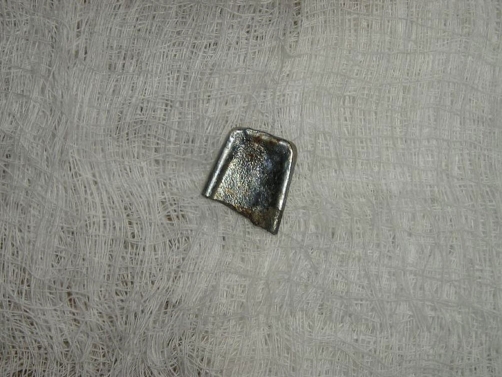
The extracted metallic foreign body.

However, he had to be referred to a neighboring Mission Hospital in Jos for proper management as the light source for a bronchoscopy in our unit was non-functional at the patient’s time of presentation. He was prepared for and had rigid bronchoscopy with the removal of a flat metallic rhomboid shaped foreign body (Figure 3) at the Mission Hospital.

He did well post-operatively on augmentin, paracetamol and vitamin c tablets. He was discharged on the second post-operative day. Follow-up of this patient was uneventful.

## Discussion

Endobronchial metallic foreign bodies are very serious injuries. The best treatment is rigid bronchoscopy [10] in order to prevent life threatening respiratory complications.The clinical presentation is that of cough, dyspnea, wheezing and fever [4], but this also depends on the type of foreign body aspirated as evidenced by the calm presentation of our patient despite showing up in hospital two days following aspiration of the foreign body. The foreign body aspirated by our patient was a flat rhomboid shaped metallic object which was lying vertically in the right main bronchus and still allowed air into the lungs without complete obstruction.

Our patient had plain X-rays which revealed the metallic foreign body, further highlighting the importance of plain radiographs in diagnosing aspirated metallic foreign bodies. Endobronchial foreign bodies can be very difficult to remove depending on the type and location of the foreign body (they preferentially lodge in the right main bronchus), the experience of the bronchoscopist and the availability of the appropriate instruments for their removal [8].

While techniques are being refined for the diagnosis and treatment of endobronchial foreign bodies [6,7], Nigeria is still grappling with the non-availability of even the basic instruments to manage such conditions. This is because our government-run hospitals are ill-funded and ill-equipped. Our patient suffered the brunt of the aforementioned with referrals between three major hospitals before receiving appropriate treatment.

Quick diagnosis and treatment of endobronchial foreign bodies is of utmost importance to avoid life threatening respiratory sequelae which include atelectasis, pneumonia, pulmonary hyperinflation and pneumomediastinum [5], a line of management that should have been instituted at the first or second hospital patient presented if properly equipped.

This therefore is a clarion call to our leaders to adequately fund and equip our hospitals to prevent such management difficulties and alleviate our people’s sufferings both young and old. Parents and guardians should ensure that dangerous objects are kept away from their children and wards in order to prevent such injuries.

## Conclusion

Our leaders should adequately fund and equip our hospitals for the management of this otherwise straight forward case and alleviate the sufferings of our people. Parents and guardians should exercise caution in the handling of their children/wards.
